# Acute Promyelocytic Leukemia in a Patient With Sickle Cell Hemoglobin D Disease: A Case Report

**DOI:** 10.7759/cureus.87513

**Published:** 2025-07-08

**Authors:** Mahmoud Bokhary, Esra Turak, Rehab Ibrahim, Aly Rashed, Mohamed Abdelhadi, Cigdem Ozturk, Shruti Sudha, Leylagul Kaynar, Elias Fadel

**Affiliations:** 1 Hematology, Sheffield Teaching Hospitals NHS Foundation Trust, Sheffield, GBR; 2 Hematology, King Hamad University Hospital, Al Muharraq, BHR

**Keywords:** acute promyelocytic leukemia (apml), deep vein thrombosis (dvt), d-punjab, hydroxycarbamide, sickle cell disease (scd)

## Abstract

Myeloid neoplasms can occur in patients with sickle cell disease; however, the underlying connection between the two remains unclear. These neoplasms, along with other cancers such as female breast cancer and male genital cancer, may arise de novo or secondarily due to chronic inflammation associated with sickling crises. This chronic inflammation, and its downstream effects, may lead to cytogenetic aberrations that increase the risk of developing myeloid neoplasms. Another potential risk factor is hydroxyurea, a medication commonly used to reduce the frequency of sickling crises, with some reported cases of associated myeloid malignancies. To explore this possible link, we present a case of acute promyelocytic leukemia in a patient with sickle cell hemoglobin D disease who had never received hydroxyurea.

## Introduction

Sickle cell disease is an inherited autosomal recessive hemoglobin disorder caused by a point mutation at the sixth codon of the β-globin gene, leading to the substitution of hydrophilic glutamic acid with hydrophobic valine. This abnormal hemoglobin is poorly soluble in its deoxygenated state [1]. The resulting pathophysiology gives rise to a range of clinical manifestations, including vaso-occlusive crises, hemolytic anemia, acute chest syndrome, and bone necrosis, complications that may lead to lifelong disability or even death [2]. Individuals who are homozygous for hemoglobin S often exhibit severe clinical symptoms, whereas heterozygous carriers typically present with minimal or no symptoms [3].

Variant sickle cell syndromes arise when hemoglobin S is inherited alongside another specific β-globin gene mutation, such as those involving the alpha, beta, or gamma globin chains. This results in compound heterozygous hemoglobinopathies that may present with clinical features similar to, or distinct from, homozygous hemoglobin S disease. One such rare variant is sickle cell hemoglobin D disease [4]. The most well-known hemoglobin D variant is HbD Los Angeles (also known as HbD Punjab), which results from a glutamic acid to glutamine substitution at codon 121 of the β-globin gene (β121 Glu→Gln) [5].

Although individuals with heterozygous mutations may be asymptomatic, those with compound heterozygosity for HbS and HbD (HbSD disease) can experience severe symptoms resembling those of homozygous HbS disease. This is due to the polymerization of HbS under deoxygenated conditions [5,6]. HbD Los Angeles shares electrophoretic mobility with HbS under alkaline conditions but can be differentiated by acidic electrophoresis or isoelectric focusing. Common clinical presentations include hemolytic anemia with splenomegaly, vaso-occlusive crises, and acute chest syndrome. Peripheral blood (PB) smears often reveal marked anisocytosis, poikilocytosis, target cells, and sickled red blood cells (RBCs) [7].

Hydroxyurea is a well-established supportive therapy for sickle cell disease. It improves RBC deformability, modifies the RBC membrane, and increases the proportion of fetal hemoglobin (HbF) [8], thereby reducing HbS levels and alleviating symptoms. However, some cases of myeloid malignancies and myelodysplastic syndromes have been reported in patients undergoing long-term hydroxyurea treatment [9,10].

Here, we report a case of a patient with sickle cell hemoglobin D disease and a history of acute chest syndrome and bone necrosis, who presented with veno-occlusive syndrome and an elevated blast count. The patient was diagnosed with a variant form of acute promyelocytic leukemia and had no prior exposure to hydroxyurea [11].

## Case presentation

A 48-year-old female patient with a known history of sickle cell disease, though the presence of a variant hemoglobin was unknown, presented with dyspnea, lower back pain, and right leg pain for one week.

Initial laboratory investigations are shown in Table 1. Liver and kidney function tests were unremarkable. The laboratory findings suggested that the patient most likely had acute leukemia and was at high risk of developing disseminated intravascular coagulation (DIC).

**Table 1:** Initial blood investigations showing leukocytosis, anemia, thrombocytopenia, and coagulopathy

Parameter	Result	Reference range	Interpretation
White blood cells	211 × 10⁹/L	4-11 × 10⁹/L	Raised
Hemoglobin	6.2 g/dL	12-16 g/dL	Low
Platelet count	74 × 10⁹/L	150-450 × 10⁹/L	Low
Reticulocyte percentage	3.60%	0-3%	Raised
Lactate dehydrogenase	1628 U/L	250-650 U/L	Raised
D-dimer	26 mg/L	0.09-0.33 mg/L	Raised
Fibrinogen	122 mg/dL	217-496 mg/dL	Low
Prothrombin time	15.5 seconds	10.7-13.9 seconds	Raised
International normalized ratio	1.33	0.61-1.17	Raised
Partial thromboplastin time	45.9 seconds	28.1-42.9 seconds	Raised

**Figure 1:**
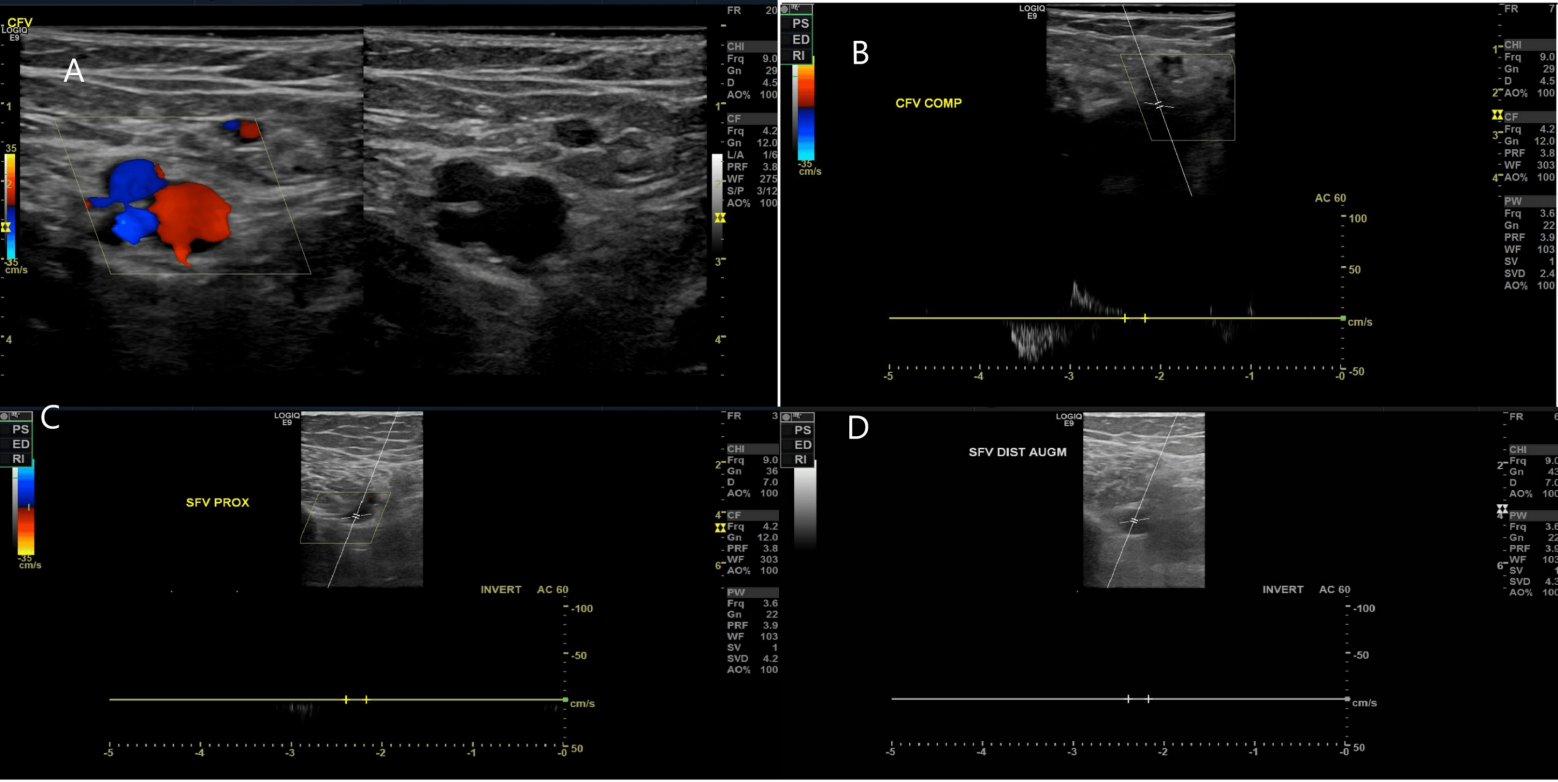
(A, B) Deep vein thrombosis in the common femoral vein demonstrated by compression test. (C, D) Deep vein thrombosis in the superficial femoral vein

A venous Doppler ultrasound of the lower limbs revealed a thrombus in the right superficial femoral vein, along with partial thrombosis of the right common femoral vein, as shown in Figure 1. Given the risk of DIC and extensive thrombosis, an inferior vena cava filter was placed.

**Figure 2:**
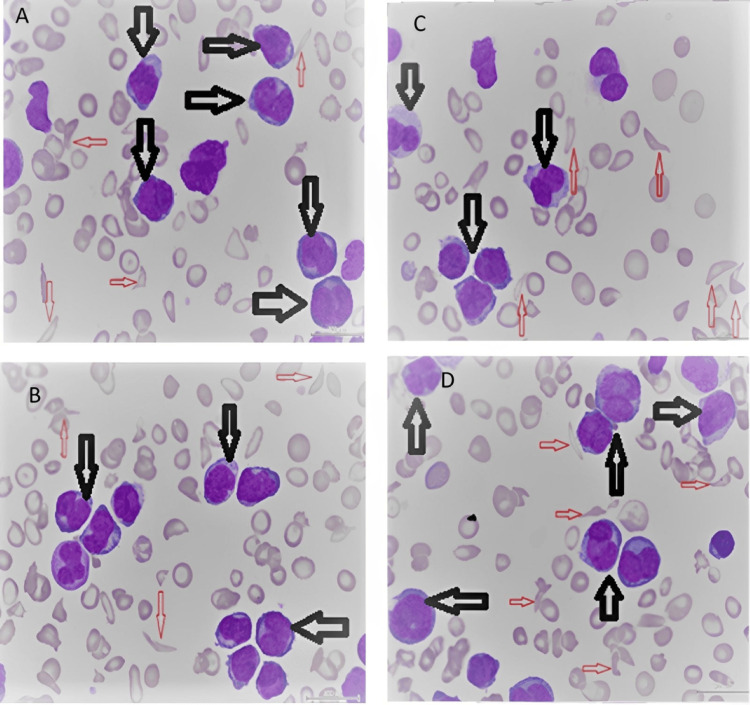
PB smear showing some sickle-shaped RBCs (red arrows) and numerous blast cells (black arrows) characterized by a low nuclear-to-cytoplasmic ratio, convoluted and dumbbell-shaped nuclei with stippled chromatin, 1-3 nucleoli per nucleus, and redundant basophilic, hypogranular cytoplasm PB, peripheral blood; RBC, red blood cell

The PB smear (Figure 2) showed marked leukocytosis with near-total infiltration by blast cells and promyelocytes. The promyelocytes appeared atypical and were suggestive of a variant promyelocytic subtype. Additionally, the smear revealed some sickle cells, a few schistocytes, teardrop cells, and occasional normoblasts.

PB flow cytometry revealed that 94.1% of the cells were CD45-positive blasts, expressing CD117, CD33, CD13, and CD64, with partial positivity for HLA-DR (40.0%). These cells were negative for CD34, MPO, TdT, CD11b, CD14, CD15, and CD16.

**Figure 3:**
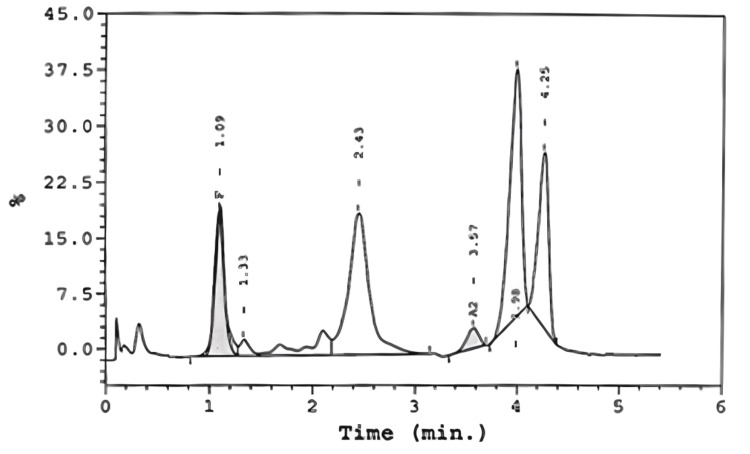
High-performance liquid chromatography (hemoglobin electrophoresis) graph demonstrating elevated levels of abnormal HbS (18.8%) and HbD (31.1%), along with an increased HbF level (14.4%), consistent with HbS/D disease and HPFH HbF, fetal hemoglobin; HPFH, hereditary persistent fetal hemoglobin

The sickling test was positive. Hemoglobin electrophoresis performed via high-performance liquid chromatography showed HbS at 18.8%, HbD at 31.1%, and an elevated HbF level at 14.4%, consistent with HbS/D trait and hereditary persistence of HbF (Table 2, Figure 3).

**Table 2:** Hemoglobin analysis showing HbF concentration of 14.4%, D-window of 31.1%, and S-window of 18.8%, indicating the presence of HbS/D disease with HPFH HbF, fetal hemoglobin; HPFH, hereditary persistent fetal hemoglobin

Peak name	Calibrated area (%)	Area (%)	Retention time (min)	Peak area
F	14.40%	-	1.09	71,563
P2	-	1.60%	1.33	7,929
Ao	-	31.20%	2.43	153,716
A2	2.80%	-	3.57	13,541
D-window	-	31.10%	3.98	153,196
S-window	-	18.80%	4.25	92,438

The initial diagnosis was acute promyelocytic leukemia (variant subtype) with concurrent deep venous thrombosis. The patient was transferred to the intensive care unit for close monitoring and to initiate chemotherapy as soon as possible. Oxygen saturation was maintained above 95% on room air.

Fluorescence in situ hybridization testing for PML-RARA returned positive, confirming the diagnosis of variant acute promyelocytic leukemia. Induction chemotherapy was initiated with cytarabine for seven days and daunorubicin for three days, alongside all-trans retinoic acid (ATRA). Therapeutic low molecular weight heparin was also started [10]. The neutropenic phase progressed without major complications, aside from a neutropenic fever, which was managed with empirical antibiotics and antifungals. The patient received RBC and platelet transfusions according to standard guidelines. Cryoprecipitate was administered to maintain fibrinogen levels above 100 mg/dL.

A follow-up Doppler ultrasound of the lower limbs after six weeks showed patent veins and complete resolution of the thrombus. Bone marrow recovery was noted, and follow-up bone marrow aspiration after induction was suggestive of morphological remission with myeloid hyperplasia, likely reflecting therapy-induced marrow regeneration. DIC had also resolved. Repeat PML-RARA testing was negative, indicating molecular remission.

The patient continued on a consolidation protocol with ATRA and arsenic trioxide and was followed closely without active complications. Rivaroxaban was introduced as an oral anticoagulant and was discontinued upon completion of consolidation chemotherapy.

## Discussion

We describe the development of a variant form of acute promyelocytic leukemia in a patient with a known history of sickle cell hemoglobin D disease - a rare condition that, to our knowledge, has not been previously reported. The underlying reason for the coexistence of myeloid neoplasms and sickle cell disorders remains unclear, though several theories have been proposed.

First, the leukemia may arise de novo, with or without a preexisting chromosomal abnormality. Second, it could result from chronic inflammatory conditions, such as bone necrosis or complications related to vaso-occlusive or hemolytic crises. Third, prolonged use of hydroxyurea has also been implicated [9,12].

In two large studies involving more than 6,000 patients with sickle cell disease, the incidence of myeloid neoplasms was reported to be 0.18%. However, the exact etiology of myeloid malignancies in these cases could not be determined [13,14].

Chromosomal analyses in previously reported cases of coexistent sickle cell disease and myeloid malignancies revealed complex cytogenetic abnormalities, including TP53 mutations, deletions of 5q-, 7q-, 17p-, trisomy 8, and KMT2A rearrangements [15]. These abnormalities are known to increase the risk of secondary malignancies regardless of the patient’s underlying medical condition [16,17]. Notably, none of these chromosomal changes were present in our patient.

Vaso-occlusive and hemolytic crises are commonly managed with repeated blood transfusions, which can lead to iron deposition in hematopoietic tissues, increased production of reactive oxygen species, and the emergence of genetic aberrations [18]. Our patient had a documented history of hemolytic crises and multiple transfusions. The presence of splenomegaly further supports prior episodes of hemolysis.

Such crises may also trigger cytokine hyperstimulation within the bone marrow, promoting disordered hematopoietic cell development as the marrow attempts to compensate for cell lysis. Chronic inflammatory processes may induce genomic instability in somatic cells, potentially leading to oncogenic mutations and the development of secondary malignancies, including both myeloid and solid tumors. Our patient had a history of bone osteonecrosis and sclerosis, along with acute chest syndrome - all indicative of chronic inflammation [3,13].

Hydroxyurea is an established treatment for sickle cell disease, known to significantly reduce the frequency of pain crises, decrease transfusion needs, lower mortality, and improve quality of life [19]. Although not classified as cytotoxic to humans by the International Agency for Research on Cancer [20], some evidence from both human and animal studies suggests a potential carcinogenic effect. Hydroxyurea inhibits ribonucleotide reductase, which may lead to an imbalance in deoxyribonucleotide pools and consequent DNA damage. These disruptions can result in genotoxic effects such as DNA strand breaks and micronuclei formation. However, our patient had never been treated with hydroxyurea, and thus, this agent is not considered a contributing factor in this case.

## Conclusions

The association between myeloid malignancies, whether acute leukemias or myelodysplastic syndromes, and sickle cell disease remains uncertain and warrants further investigation. Several possible risk factors have been identified, including prolonged hydroxyurea use, preexisting cytogenetic abnormalities, and somatic mutations driven by chronic inflammation. Repeated hemolytic episodes can lead to bone marrow stress and cytokine release, promoting compensatory hematopoiesis and potentially resulting in disordered cell development. Further research involving larger patient cohorts is necessary to clarify the risk factors contributing to secondary myeloid malignancies in individuals with sickle cell disease.
